# Trends in surgical management of spinal metastases in a Singaporean tertiary referral center: a 17-year retrospective review

**DOI:** 10.3389/fonc.2023.1297553

**Published:** 2023-11-22

**Authors:** Jiong Hao Jonathan Tan, James Thomas Patrick Decourcy Hallinan, Renick Lee, Yiong Huak Chan, Tuan Hao Tan, Shi Wei Ang, Le Tian Isaac Tan, Joelle Hwee Inn Tan, Qinxiang Shant Sin, Dennis Hwee Weng Hey, Leok Lim Lau, Joseph Thambiah, Hee Kit Wong, Gabriel Liu, Naresh Kumar

**Affiliations:** ^1^Department of Orthopedic Surgery, National University Health System, Singapore, Singapore; ^2^Department of Diagnostic Imaging, National University Hospital, Singapore, Singapore; ^3^Department of Diagnostic Radiology, Yong Loo Lin School of Medicine, National University of Singapore, Singapore, Singapore; ^4^Biostatistics Unit, Yong Loo Lin School of Medicine, Singapore, Singapore; ^5^Yong Loo Lin School of Medicine, National University of Singapore, Singapore, Singapore

**Keywords:** spinal metastases, vertebral metastases, epidural spinal cord compression, metastatic epidural spinal cord compression, minimally invasive spinal surgery, separation surgery, spinal instability

## Abstract

**Introduction:**

Surgical treatment is increasingly the treatment of choice in cancer patients with epidural spinal cord compression and spinal instability. There has also been an evolution in surgical treatment with the advent of minimally invasive surgical (MIS) techniques and separation surgery. This paper aims to investigate the changes in epidemiology, surgical technique, outcomes and complications in the last 17 years in a tertiary referral center in Singapore.

**Methods:**

This is a retrospective study of 383 patients with surgically treated spinal metastases treated between January 2005 to January 2022. Patients were divided into 3 groups, patients treated between 2005 – 2010, 2011-2016, and 2017- 2021. Demographic, oncological, surgical, patient outcome and survival data were collected. Statistical analysis with univariate analysis was performed to compare the groups.

**Results:**

There was an increase in surgical treatment (87 vs 105 vs 191). Lung, Breast and prostate cancer were the most common tumor types respectively. There was a significant increase in MIS(p<0.001) and Separation surgery (p<0.001). There was also a significant decrease in mean blood loss (1061ml vs 664 ml vs 594ml) (p<0.001) and total transfusion (562ml vs 349ml vs 239ml) (p<0.001). Group 3 patients were more likely to have improved or normal neurology (p=<0.001) and independent ambulatory status(p=0.012). There was no significant change in overall survival.

**Conclusion:**

There has been a significant change in our surgical practice with decreased blood loss, transfusion and improved neurological and functional outcomes. Patients should be managed in a multidisciplinary manner and surgical treatment should be recommended when indicated.

## 1 Introduction

The spine is the most common site of bony metastases with up to 50% of all bony metastases involving the spine (1). It is estimated that 10-20% of all patients with cancer develop symptomatic spinal metastases. The common presentations of spinal metastases include neurological deficits due to spinal cord compression and axial/radicular pain due to spinal instability or a combination of both.

In 2005, Patchell et al. (2) published a landmark randomized control trial which revolutionized the treatment of patients with metastatic epidural spinal cord compression (MESCC). This study showed that MESCC patients treated with surgery followed by radiotherapy were more likely to walk after treatment, walk longer after treatment and require less corticosteroids or opiates. This study led to a paradigm shift towards surgical management, prior to which these patients were typically treated with radiotherapy alone (3).

Spinal instability secondary to bone loss due to tumor involvement or pathological fractures is not amenable to treatment with radiotherapy or systemic therapy alone. The Spinal Instability in Neoplasia Score (SINS) was developed in 2010 to allow surgeons to better diagnose and classify spinal instability (4). The SINS is made up of 5 radiologic components, the location of spinal metastases, the nature of bone lesion, radiographic spinal alignment, amount of vertebral body collapse and amount of posterolateral element involvement, as well as one clinical component, the nature of pain which the patient is experiencing. A SINS score of 13-18 denotes spinal instability, 7-12 indeterminate stability and 1-6 normal spinal stability. Huisman et al. (5) showed that a higher SINS score was associated with failure of radiotherapy, while Hussain et al. (6) showed that patients with moderate or high SINS scores experienced significant improvement in pain and functional outcomes after surgical stabilization. Spinal stabilization for patients with spinal instability alone has become an increasingly accepted part of management of patients with spinal metastases (7, 8).

In this time period there has also been an evolution in surgical technique. Minimally invasive spine surgery in the form of percutaneous pedicle screw fixation and mini-open approaches for spinal decompression have been shown to reduce blood loss, surgical complication rate and duration of hospital stay (9, 10). The advent of stereotactic radiotherapy (SBRT) has led to increased acceptance of the concept of separation surgery where the goal of surgery is to create a 1-2mm area of separation between the tumor and the spinal cord to allow for safe SBRT (11), rather than achieving gross total tumor removal with a corpectomy or spondylectomy. In appropriately selected patients where there is an appropriate margin after separation surgery between the spinal cord and tumor local recurrence rates of less than 10% have been reported (12). Intra and perioperative management have also evolved with the use of intra-operative neuromonitoring to detect intraoperative and prevent post-operative neurological deficit (13) as well as intraoperative cell salvage and autogenic blood transfusion to reduce the need for allogenic blood transfusion (14).

This paper aims to investigate the changes in epidemiology, surgical technique, outcomes and complications in the last 17 years in a tertiary referral center in Singapore.

## 2 Materials and methods

This is a retrospective study of 383 patients with surgically treated spinal metastases at a Singaporean tertiary referral center between January 2005 to January 2022. We obtained institutional review board (IRB) approval prior to starting this study.

All patients were aged 18 and above. Indications for surgery included MESCC and/or spinal instability. We excluded patients who had primary spine tumors, patients who had previous spinal surgery for spinal metastases and other spinal conditions, and patients who have undergone vertebroplasty, kyphoplasty or other interventional procedures.

Patients were divided into 3 groups, patients treated between 2005 January and 2010 January, between 2011 January and 2016 January, and between 2017 January and 2022 January. This was labelled Group 1, Group 2 and Group 3 respectively. Basic demographic data was collected including age at time of surgery, race, gender, pre-operative Eastern Cooperative Oncology Group (ECOG) score and Charlson Comorbidity Index (CCI). Oncological data collected included primary tumor histology, tumor subtype according to the modified Tokuhashi score (15), number of vertebral metastases, number of spinal metastases, number of visceral metastases, pre-operative Karnofsky Performance score and pre-operative Frankel score. The Frankel score was categorized into 3 categories, Frankel A+B (No motor function), Frankel C+D (motor function present but abnormal) and Frankel E (normal motor function). The modified Tokuhashi score was calculated based on the above data. Spinal metastases location was also recorded.

The decision for surgical treatment was a multidisciplinary decision involving the spinal surgeon, oncologist, radiation oncologist and other members of the multidisciplinary team. The patients neurological status, based on the severity of cord compression and Frankel score, spinal stability based on the SINS, oncological status based on radiation oncologist and oncologist opinion, and systemic status based on the ECOG and Karnofsky score, Charlson Comorbidity Index (CCI) and prognosticated survival based on the modified Tokuhashi score were all taken into account. A thorough history and examination is performed to determine the symptomatic levels. Only the symptomatic levels, levels at which iatrogenic instability is expected, and indeterminate stability or unstable based on the SINs score are instrumented. Where there is a vertebral level of indeterminate stability adjacent to an instrumented level the decision whether to include it within the construct is a multidisciplinary decision based on the sensitivity of the tumor to radiotherapy, systemic treatment, and the surgeon’s assessment of stability.

Surgical data collected included surgical approach, which was divided into posterior, anterior and combined approaches. A posterior approach is defined as either the utilization of a midline longitudinal posterior open approach to the spine or a minimally invasive percutaneous approach for insertion of posterior pedicle screws in the thoracolumbar spine. This is indicated in stabilization only, stabilization and decompression and separation surgery. It can also be utilized for partial or subtotal corpectomy of the thoracolumbar spine. Non-posterior approaches include anterior Smith Robinson type approach to the cervical spine, lateral retroperitoneal approach to the lumbar spine and thoracotomy approach to the thoracic spine. Surgical technique was divided into open, minimally invasive or hybrid techniques. Anterior cervical procedures were all classified as open procedures. Surgical type was divided into stabilization only, posterior decompression and stabilization, separation surgery, partial and complete corpectomy. Stabilization procedures were defined as those where only posterior instrumentation with screws and rods were performed. Posterior decompression and stabilization included posterior stabilization with screws followed by posterior laminectomy with no attempt at circumferential decompression. Separation surgery was one where a laminectomy was performed followed by anterior decompression via a transpedicular approach for circumferential tumor decompression, with no attempt made at anterior reconstruction. This was performed as an open technique as described by Laufer et al. (12), or as a mini-open technique as described by Kumar et al. (9). A partial corpectomy was a piecemeal intralesional excision of up to 70% of the vertebral body while a near total corpectomy was one where more than 70% of the vertebral body was excised followed by an anterior column reconstruction. In our study group partial or total corpectomies were performed for the following groups of patients. In patients with cervical three to thoracic one vertebral body involvement with MESCC where separation surgery from a posterior approach would be challenging and less commonly in patients with non-radiosensitive tumors with thoracolumbar involvement and good survival prognosis (≥1 year) and good functional status (ECOG 0-1). Fusion was performed when a partial or subtotal corpectomy is performed. We do not perform fusion when stabilization only, posterior decompression and stabilization and separation surgery are performed. We also recorded the number of levels decompressed, the number of levels instrumented and the need for pre-operative angioembolization.

Intra-operative blood loss was calculated based on the estimation of the surgical and anesthetic team. Volume of allogenic and autogenic blood transfusion were recorded. Duration of stay and duration of High Dependency/Surgical Intensive Care Unit (HD/SICU) stay were recorded. Post-operative outcome in terms of post-operative Frankel Score and post-operative ambulatory status were also recorded. Complications requiring surgical or medical management were noted and need for readmission within 1-month post discharge was also recorded.

Duration of survival was defined as time from surgery to ultimate demise of patient. 1-month, 3-months, 6-months and 12-months survival were also recorded. The actual survival of each patient was compared to survival duration predicted by the modified Tokuhashi score and we recorded if actual survival was shorter, longer or the same as the prognosticated survival.

Statistical Analysis was performed with the use of SPSS Statistical Software (IBM SPSS Statistics Version 28). Two Sample T test/Mann Whitney U test were used to compare the differences for numerical variables between the two age groups and the Pearson Chi Squared test or Fishers Exact test were used for categorical variables. Overall survival between the two groups was presented with a Kaplan-Meier analysis. A p value of <0.05 was taken to be significant.

## 3 Results

A total of 412 patients were analyzed of which 383 patients met the criteria for inclusion. There was an increase in number of patients being operated in each consecutive group with there being 2.2 times the number of patients operated in Group 3 when compared to Group 1 (191 vs 87 patients). There was no significant difference in age, sex, race and Charlson Comorbidity Index of patients among all 3 time periods. Group 2 patients had significantly worse pre-morbid ECOG score when compared to the other two time periods (p=0.003). This data is shown in **Table 1**. In our study, lung cancer (110 (28.7%) patients), followed by breast cancer (75 (19.6%) patients) and prostate cancer (32 (8.4%) patients) respectively were consistently the most common primary tumors throughout the duration of this study. There was no significant difference in modified Tokuhashi tumor subtype among all 3 groups. There was a significant difference in pre-operative Frankel score with 49.7% of Group 3 patients being Frankel E as compared to 40% in Group B and 23% in Group A. Patients operated between 2011-2016 had significantly less visceral metastases (p=0.008). There was no significant difference in number of patients in each Modified Tokuhashi score category, number of vertebral metastases and number of extra spinal metastases between the 3 groups. Oncological data is shown in **Table 2**.

**Table 1:** Baseline demographic characteristics of patients.

Parameter	All Patients	2005-2010	2011-2016	2017-2021	p value
Number of patients	n= 383	n=87	n=105	n=191	
Mean age, (years)(range)	61(26-87)	58 (28-87)	62 (26-86)	63 (28-86)	
Sex					
Male	184 (48%)	39 (44.8%)	55 (52.4%)	90 (47.1%)	
Female	199 (52%)	48 (55.2%)	50 (47.6%)	101(52.9%)	
Race					
Chinese	273 (71.3%)	63 (74.1%)	71 (67.6%)	139(72.8%)	
Malay	57 (14.9%)	11 (12.9%)	18 (17.1%)	28(14.7%)	
Indian	15 (3.9%)	4 (4.7%)	5 (4.8%)	6 (3.1%)	
Other	36 (9.4%)	7 (8.2%)	11 (10.5%)	18 (9.4%)	
Preoperative ECOG Score					
0-2	345 (90.1%)	82 (94.3%)	83 (83.0%)	155(94.5%)	0.003
3-4	38 (9.9%)	5 (5.7%)	17 (17.0%)	9(5.5%)	
Charlson Comorbidity Index	8 (2-15)	8 (2-12)	8 (2-12)	8 (2-15)	

ECOG, Eastern Cooperative Oncology Group.

**Table 2:** Oncological characteristics of patients.

Parameter	All Patients	2005-2010	2011-2016	2017-2021	p value
Tumor Histology					
Lung	110 (28.7%)	22(25.3%)	30 (28.6%)	58(28.6%)	
Breast	75 (19.6%)	15 (17.2%)	21 (20.0%)	39(20.0%)	
Prostate	32 (8.4%)	8 (9.2%)	8 (7.6%)	16(8.4%)	
Multiple Myeloma	27 (7.0%)	8 (9.2%)	4 (3.8%)	15 (7.9%)	
Renal	25 (6.5%)	5 (5.7%)	8 (7.6%)	12 (6.3%)	
Colorectal	19 (5.0%)	6 (6.9%)	4 (3.8%)	9 (4.7%)	
Liver	16 (4.2%)	2 (2.3%)	7 (6.7%)	7 (3.7%)	
Lymphoma	16 (4.2%)	7 (8.0%)	6 (5.7%)	3 (1.6%)	
Tumor subtype					
0	132(34.5%)	25 (28.7%)	36(34.3%)	71(37.2%)	
1	24 (6.3%)	3(3.4%)	8 (7.6%)	13(6.8%)	
2	69 (18.0%)	23 (26.4%)	18 (17.1%)	28 (14.7%)	
3	26 (6.8%)	5(5.7%)	10 (9.5%)	11 (5.8%)	
4	20 (5.2%)	5(5.7%)	4 (3.8%)	11(5.8%)	
5	112 (29.2%)	26(29.9%)	29 (27.6%)	57 (29.8%)	
Number of Vertebral Metastases					
1	74 (19.4%)	18 (20.7%)	22 (21.0%)	34 (18.0%)	
2	76 (19.9%)	17(19.5%)	13 (12.4%)	46 (24.3%)	
≥3	231 (60.6%)	52(59.8%)	70 (66.7%)	109 (57.7%)	
Number of Extra-Spinal					
Metastases	151 (39.4%)	33 (37.9%)	46 (43.8%)	72(38.1%)	
0	92 (24.0%)	20 (23.0%)	20 (19.0%)	52 (27.5%)	
1-2	138 (36.0%)	34 (39.1%)	39 (37.1%)	65 (34.4%)	
≥3					
Visceral Metastases					
None	168 (44.1%)	32(36.8%)	55(52.4%)	81(42.9%)	0.008
Removable	14 (3.7%)	3 (3.4%)	8 (7.6%)	3(1.6%)	
Unremovable	199 (52.2%)	52 (59.8%)	42(40.0%)	105 (55.6%)	
Pre-operative Frankel Score					
Complete (Frankel A or B)	16 (4.2%)	5 (5.7%)	4 (3.8%)	7 (3.7%)	
Incomplete (Frankel C or D)	209 (54.9%)	62 (71.3%)	59 (56.2%)	88 (46.6%)	
Normal (Frankel E)	156 (40.9%)	20 (23.0%)	42(40.0%)	94 (49.7%)	0.001
Modified Tokuhashi Score					
0-8	213 (55.9%)	52(59.8%)	58 (55.2%)	103 (54.5%)	
9-11	138 (36.2%)	27 (31.0%)	41 (39.0%)	70 (37.0%)	
12-15	30 (7.9%)	8 (9.2%)	6 (5.7%)	16 (8.5%)	

The posterior approach was the most common surgical approach employed in all 3 groups (340 (91.4%) patients), followed by combined (25 (6.7%)) and anterior approaches (7 (1.9%)). In the first group of patients, minimally invasive techniques were not performed, but in the subsequent 2 groups, MIS techniques, for 45/105 (43.3%) patients in Group 2 and 64/191 (35.4%) patients in Group 3, became significantly increasingly adopted (p<0.001). There was no significant difference in number of levels instrumented between the time periods, but patients in Group 1 and Group 3 required significantly more levels of decompression (p=0.01). Surgical characteristics are shown in **Table 3**.

**Table 3:** Surgical characteristics of patients.

Parameter	All Patients	2005-2010	2011-2016	2017-2021	p value
Location of Tumor					
Cervical	22 (5.7%)	4 (4.6%)	6 (5.7%)	12 (6.3%)	
Cervicothoracic Junction	33 (8.6%)	3 (3.5%)	9 (8.6%)	21 (11.0%)	
Thoracic	167 (43.6%)	44 (50.6%)	40 (38.1%)	83 (43.5%)	
Thoracolumbar Junction	56 (14.6%)	12 (13.8%)	17 (16.2%)	27 (14.1%)	
Lumbar	67 (17.5%)	15 (17.2%)	26 (24.8%)	26 (13.6%)	
Lumbosacral Junction	11 (2.9%)	4 (4.6%)	1 (0.9%)	6 (3.1%)	
Sacral	3 (0.8%)	1 (1.2%)	0 (0.0%)	2 (1.1%)	
Multiple	24 (6.3%)	5 (5.7%)	6 (5.7%)	13 (6.8%)	
Type of Surgery					
Stabilization only	71(19.1%)	11(12.8%)	33(31.7%)	27 (14.9%)	
Stabilization and decompression	144 (37.6%)	57 (66.3%)	42 (40.4%)	45 (24.9%)	
Separation Surgery	98 (26.4%)	6 (7.0%)	15 (14.4%)	77 (42.5%)	<0.001
Partial Corpectomy	52 (13.6%)	9 (10.5%)	14 (13.5%)	29 (16.0%)	
Complete Corpectomy	6 (1.6%)	3(3.5%)	0 (0.0%)	3 (1.7%)	
Surgical Approach					
Posterior	340 (91.4%)	78 (90.7%)	95(91.3%)	167 (91.8%)	
Anterior	7 (1.9%)	0 (0.0%)	2 (1.9%)	5 (2.7%)	
Combined	25 (6.7%)	8(9.3%)	7 (6.7%)	10 (5.5%)	
Surgical Technique					
Open	249 (67.1%)	86 (100%)	56(53.8%)	107 (59.1%)	
Minimally invasive surgery	109 (29.4%)	0 (0.0%)	45(43.3%)	64 (35.4%)	<0.001
Hybrid	13 (3.5%)	0 (0.0%)	3 (2.9%)	10 (5.5%)	
Number of levels Instrumented Mean (range)	7(0-17)	7(0-14)	7(0-17)	6(0-16)	
Number of levels Decompressed Mean (range)	2(0-7)	2(0-6)	1(0-6)	2(0-7)	p=0.01
Pre-operative Angioembolization					
Yes	59(15.4%)	15 (17.2%)	20 (19.0%)	24 (12.6%)	
No	324 (84.6%)	72 (82.8%)	85 (81.0%)	167 (87.4%)	

Mean blood loss significantly decreased over the course of this study (Group 1 vs Group 2 vs Group 3) (1061ml vs 664 ml vs 594ml) (p<0.001) and there was also a significant decrease in mean total transfusion (562ml vs 349ml vs 239ml) (p<0.001). Autogenic blood transfusion via a cell saver was increasingly performed with 47/191 (25.1%) patients in group 3 receiving autogenic blood transfusion compared to 8/105 (7.6%) patients in group 2 and no patients in group 1. There was a significant difference in post-operative neurological outcome (p=<0.001) and ambulatory status (p=0.012). 163/191 (85.3%) of Group 3 patients had an improvement in or maintained normal neurology, as compared to 69/105 (65.7%) of Group 2 patients and 43/87 (49.4%) of Group 1 patients. Patients in Group 3 were more likely to walk independently, 110/191 (57.6%) vs 48/105 (45.7%) in Group 2 and 33/87 (37.9%) patients in Group 1. However, there were significantly more surgical complications in the Group 2 patients (p=0.025). Patient outcomes are shown in **Table 4**.

**Table 4:** Perioperative and postoperative outcomes.

Parameter	All Patients	2005-2010	2011-2016	2017-2021	p value
Blood Loss mls(mean)(range)	720 (10-6400)	1061 (200-5500)	664 (20-3500)	594 (10-6400)	<0.001
Total Transfusion mls(mean)(range)	340 (0-5500)	562 (0-3500)	349 (0-2730)	239 (0-5500)	<0.001
Type of Blood Transfusion					
No Blood Transfusion	206 (55.2%)	36 (44.4%)	61 (58.1%)	109 (58.3%)	<0.001
Allogenic	109 (29.2%)	44 (54.3%)	36 (34.3%)	29 (15.5%)	
Autogenic	55 (14.7%)	0 (0.0%)	8 (7.6%)	47 (25.1%)	
Allogenic and Autogenic	3 (0.8%)	1 (1.2%)	0 (0.0%)	2 (1.1%)	
Duration of HD/SICU Stay	3 (0-36)	4 (0-20)	3(0-36)	2 (0-19)	0.026
Duration of Stay	24 (3-210)	20 (5-80)	25 (3-93)	25 (3-210)	
Post-operative Neurology					
Worsened	30 (7.8%)	8 (9.2%)	10 (9.5%)	12 (6.3%)	
No Change	78 (20.4%)	36 (41.4%)	26 (24.8%)	16 (8.4%)	
Improved or maintained normal neurology	275 (71.8%)	43 (49.4%)	69 (65.7%)	163 (85.3%)	<0.001
Ambulatory Status Bedbound	34 (8.9%)	12 (13.8%)	12 (11.4%)	10 (5.2%)	
Wheelchair bound Walking Frame	47 (15.3%) 60 (15.7%)	8 (9.2%) 19 (21.8%)	17 (16.2%) 15 (14.3%)	22 (11.5%) 26 (13.6%)	
Walking Stick	27 (7.0%)	5 (5.7%)	6 (5.7%)	16 (8.4%)	
Ambulant with assistance/supervision	24 (6.3%)	10 (11.5%)	7 (6.7%)	7 (3.7%)	
Independent	191 (49.9%)	33 (37.9%)	48 (45.7%)	110 (57.6%)	0.012
Medical Complication					
Yes (%)	191 (49.9%)	38 (43.7%)	60 (57.1%)	93 (48.9%)	
No (%)	192 (50.1%)	49 (56.3%)	45 (42.9%)	97 (51.1%)	
Surgical Complication					
Yes (%)	79 (20.7%)	13 (14.9%)	31 (29.5%)	35 (18.4%)	0.025
No (%)	303 (79.3%)	74 (85.1%)	74 (70.5%)	155 (81.4%)	
Readmission within 1 month					
Yes (%)	103 (30.4%)	22 (29.0%)	34 (36.6%)	47 (27.7%)	
No (%)	236 (69.6%)	54 (71.0%)	59 (63.4%)	123 (72.3%)	

HD/SICU, High Dependency/Surgical Intensive Care Unit.

**Figure 1:**
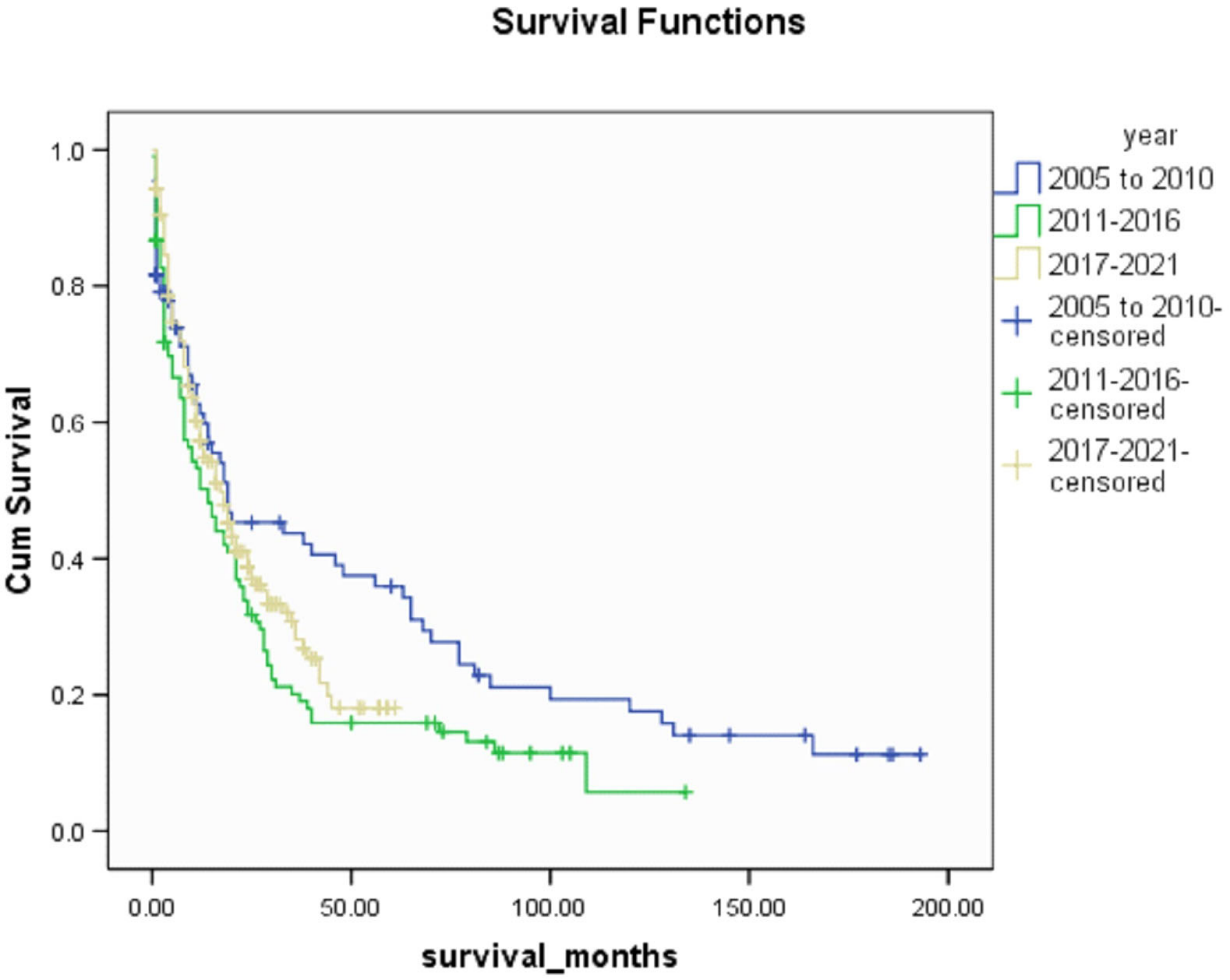
Kaplan Meir Curve comparing survival between 3 groups (2005-2010 vs 2011-2016 vs 2017-2021).

Median survival was 16(0-193) months in this cohort and there was no significant difference in 12-months and 6-months survival. There was a significant increase in 3-months and 1-month survival when compared to the 2005-2010 group of patients (p<0.005). 53.5% and 54.5% of patients in 2011-2016 and 2017-2021 respectively outlived their prognosticated survival although this was not statistically significant. **Table 5** shows patient survival characteristics. A Kaplan Meir Curve comparing the survival of all 3 groups is shown on **Figure 1**.

**Table 5:** Post-operative patient survival characteristics.

Parameter	All Patients	2005-2010	2011-2016	2017-2021	p value
Median survival (months) (range.)	16(0-193)	19 (0-193)	14 (0-134)	17 (0-61)	
Survival ≥ 12 months					
Yes	200 (52.2%)	44 (50.6%)	52(49.5%)	104 (54.5%)	
No	183 (47.8%)	43 (49.4%)	53 (50.5%)	87 (45.5%)	
Survival ≥ 6 months					
Yes	258 (67.4%)	57 (65.5%)	65 (61.9%)	136 (71.2%)	
No	125 (32.6%)	30 (34.5%)	40 (38.1%)	55 (38.8%)	
Survival ≥ 3 months					
Yes	315 (82.2%)	64 (73.6%)	82 (78.1%)	169 (88.5%)	0.004
No	68 (17.8%)	23 (26.4%)	23 (21.9%)	22 (11.5%)	
Survival ≥ 1 months					
Yes	331 (86.4%)	67 (77.0%)	87 (82.9%)	177 (92.7%)	0.001
No	52 (13.6%)	20 (23.0%)	18 (17.1%)	14 (7.3%)	
Survival					
Shorter then prognosticated	44 (11.5%)	9 (11.0%)	14 (14.1%)	21 (11.0%)	
Similar to prognosticated	131 (34.2%)	33 (40.2%)	32 (32.3%)	66 (34.6%)	
Longer then prognosticated	197 (51.4%)	40 (48.8%)	53(53.5%)	104 (54.5%)	

## 4 Discussion

Singapore has an aging population, with 23.8% of the population estimated to be aged 65 and above by 2035. Cancer incidence is known to increase with age (16) and this combined with advances in oncological care leading to increased survival will lead to more patients presenting with spinal metastases. Advances in surgical techniques and increased acceptance of surgical treatment for MESCC and spinal instability secondary to metastases has led to an increase in surgical treatment of spinal metastases (3). This is reflected in our study where in Group 1 (2005-2010) 15 patients were operated per year, while in Group 3 (2017-2022) this had more than doubled to 38 patients per year.

In our cohort lung cancer (110 (28.7%) patients), breast cancer (75 (19.6%) patients) and prostate cancer (32 (8.4%) patients) were the most common primary tumors. Wright et al. (17) compared metastatic spine tumor epidemiology in centers in Asia, Europe, North America and the United Kingdom from 1991 to 2016. In their study they found that Asian centers had higher frequencies of lung, colon and liver spinal metastases and lower frequencies of breast, prostate and multiple myeloma spinal metastases. In our cohort lung cancer spinal metastases percentage was similar to that of other Asian centers, 28.7% vs 28.1%, but our percentage of breast and prostate cancer metastases were markedly higher (19.6% vs 5.9%) and (8.4% vs 4.6%) respectively and more in keeping with that of other centers worldwide. The mean age of presentation in our cohort was 61 (26-87) years, which was similar to the findings of Wright et al. (17) where the range of age of presentation was 58 to 62 years across all regions. Of note was the higher percentage of female vs male patients in our cohort (199 (52%) vs 184 (48%)) respectively, while in other centers the percentage of male patients ranged from 55-60%.

**Figure 2:**
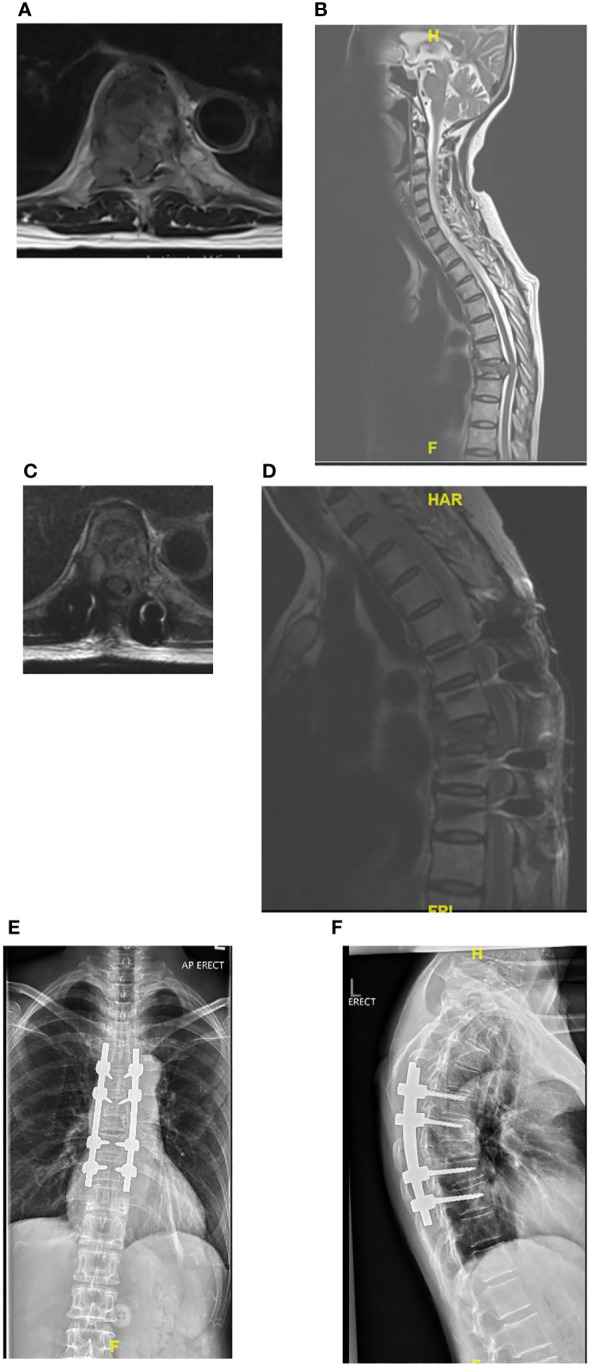
**(A–F)** Showing patient with Non-Small Cell Lung Cancer metastatic epidural spinal cord compression at T8 treated with separation surgery and no anterior reconstruction or fusion. Post-operative imaging at 2 years show no evidence of construct failure. **(A)** Pre-operative Axial MRI of T8 with Bilsky 2 cord compression. **(B)** Pre-operative T2 Sagittal MRI with cord compression at T8. **(C)** Post-operative Axial MRI of T8 showing decompression of the spinal cord. **(D)** Post-operative Sagittal MRI showing decompression at T8. **(E)** Post-operative AP radiograph at 2 years follow-up. **(F)** Post-operative lateral x-ray at 2 years.

**Figure 3:**
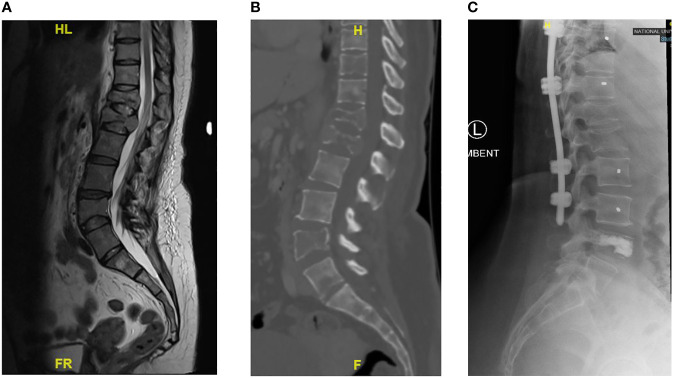
Showing patient with Breast cancer and multiple level vertebral involvement. There were pathological fractures at T12 and L1, SINs score 14 respectively. At T10, the Sins score was 11 and at L4 the Sins Score was 8. Minimally Invasive Posterior Instrumentation was performed from T9 to L3 and vertebroplasty at L4. **(A)** Pre-operative T2 Sagittal MRI showing multiple levels of vertebral involvement. **(B)** Pre-operative Lumbar CT scan showing multiple osteolytic lesions. **(C)** Post-operative Lateral radiograph.

In a review of surgical trends in the treatment of spinal metastases in the past ten years by Orenday-Barraza et al. (18), there has been an increased trend of performing separation surgery and utilizing minimally invasive techniques. When compared to posterior decompression alone, anterolateral or circumferential decompression is associated with a higher chance of neurologic improvement. Separation surgery via a posterior transpedicular approach is used to achieve circumferential decompression without the approach related complications of anterior or lateral approaches. The advent of separation surgery has led to a decrease in the use of non-posterior approaches to the thoracolumbar spine, and these approaches were no longer utilized in the 2011-2016 and 2017-2021 period. Posterior decompression and stabilization were previously the most common surgical type, with 57 (66.3%) patients in Group 1 treated this way, however its use has declined and in Group 3 this was the treatment of choice in only 24.9% of patients in our center. There has been a significant increase in the use of separation surgery to treat patients with spinal metastases from 6 (7.0%) patients in Group 1 to 77 (42.5%) patients in Group 3. An example of a case treated with separation surgery is shown in **Figure 2**. Minimally invasive techniques can be incorporated in separation surgery via the use of percutaneous pedicle screw fixation for stabilization and mini-open or tubular approaches for decompression. Kumar et al. (9) found that separation surgery via a mini-open approach with percutaneous pedicle screws compared to open posterior instrumentation and separation surgery, was associated with lower perioperative blood loss (602 mL vs 1008 mL) (P <.001), and a trend towards shorter hospital stay (10 days vs 18 days) (P = .098). There has been a significant increase in the use of minimally invasive surgical techniques compared to traditional open techniques; in 2005-2010, hybrid or minimally invasive techniques were not utilized, but in the 2017-2021 group, 64 (35.4%) patients had MIS surgery and 10 (5.5%) patients had hybrid procedures. Kumar et al. (10) showed that patients treated with percutaneous pedicle screw fixation had a significant reduction in intraoperative blood loss and time to initiate radiotherapy after surgery. In a meta-analysis by Lu et al. (19) minimally invasive spine surgery in spinal metastases was associated with a significant reduction in blood loss, length of stay, and incidence of complications. In our cohort there was a significant reduction in mean blood loss (p<0.001) and mean total blood transfusion (p<0.001), and we attribute this to the increased use of minimally invasive techniques in treating our patients. Other contributing factors include modifications in anesthetic techniques, the use of antifibrinolytic agents and preoperative embolization (20–22). A patient with multiple level vertebral involvement treated with minimally invasive instrumentation for instability is shown in **Figure 3**.

Allogenic blood transfusion is still the mainstay of treatment for intraoperative blood loss, however the demand for allogenic blood often exceeds its supply and allogenic blood transfusion is not without its complications. In a multicenter prospective review of 1601 patients by De la Garza et al. (23), patients who received a transfusion had a significantly higher complication rate when compared to non-transfused patients, (22.3% vs. 15.0%, P < 0.001) (24). These complications included sepsis, deep vein thrombosis, and prolonged ventilation. Kumar et al. (25, 26) had previously shown that a leukocyte depletion filter was able to remove tumor cells or render them non-viable, making autogenic blood transfusion via cell salvage unlikely to lead to dissemination of tumor cells and metastases. We have increasingly made use of intraoperative cell salvage and this has allowed us to avoid allogenic blood transfusion in 25.1% of patients in Group 3 (27).

Significantly more patients in group 3 maintained normal neurology or had improvements in their neurology (p<0.001) and significantly more patients were able to ambulate independently (p=0.012). This may be attributable to 2 changes in surgical practice. Firstly, we have increasingly treated patients for spinal instability secondary to spinal metastases, these patients largely have normal neurology prior to surgery and surgical treatment is to treat mechanical pain and prevent development of neurological deficits due to spinal instability. Secondly, separation surgery is increasingly practiced and this allowed anterior decompression of the neural elements which is not possible with posterior decompression alone (9). In patients who undergo separation surgery more than 90% local control rate at 1 year has been reported by Laufer et al. (28), and Cofano et al. (29) reported a significantly higher rate of neurological improvement (94.1% vs 60.4%) in patients who underwent circumferential decompression rather than posterior decompression alone. Intraoperative neuromonitoring has also become a standard of care in our practice, due to its sensitivity and specificity in detecting intraoperative neurological events and allowing the surgeon to take steps to reverse the causes of intraoperative neurological events (13).

Surgical treatment of spinal metastases is associated with a significant complication rate, in our cohort the medical complication rate was 49.9% and the surgical complication rate was 20.7%. Patients should be made aware of this as part of their pre-surgical counselling. While there was a significant increase in 1-month and 3-months survival in Group 2 and 3 (p<0.005), there was no increase in overall survival. Patient survival is influenced more by tumor histology and patient performance status, rather than surgical type or invasiveness (8). However even patients with a poor life expectancy or functional status should not necessarily be denied surgical treatment, Dea et al. (30) have found that even patients with less than 3 months life expectancy experience an improvement in quality of life at 6 weeks and Amelot et al. (31) have found that patients with ECOG scores of 3-4 can experience improvements in neurological outcome and quality of life.

The treatment of spinal metastases is a multidisciplinary endeavor requiring the consideration of multiple patient factors. Various treatment algorithms and frameworks have been developed to guide treatment of these patients (7, 32, 33). The NOMS framework was one of the first modern treatment frameworks devised (7). It considers the patients neurological, oncological, mechanical, and systemic status, and its advantages include its ease of recall and use. However, it does not explicitly state the importance of other patient factors such as the number of and location of spinal levels involved and the patient’s previous response to chemotherapy. The LMNOP framework takes into account these factors (32, 33). Its components include location and level of spinal disease; mechanical instability; neurology; oncology; patient fitness, prognosis, patient wishes and prior therapy. The location of spinal metastases in the anterior or posterior column of the spine and level of involvement directly involves surgical approach and patients with multiple levels of involvement may need extension of instrumentation or more than one procedure. Preoperative therapy and prior response are also important to consider as a patient who does not respond to previous systemic or local treatments is likely to have a poorer prognosis. While the above factors were considered prior to operative treatment in our patients, a systemic approach with use of the above framework may be beneficial in-patient care. This may also reduce the amount of variability in treatment. It is also important to take into consideration advances in therapy which may make these frameworks out of date, however the basic components of the NOMS and LMNOP framework still remain relevant.

Limitations of this study include the retrospective nature of the study and lack of information on preoperative and postoperative systemic therapy and response to systemic therapy of these patients. We were thus unable to assess the effect of prior systemic therapy on the 3 groups of patients especially on survival. Another limitation is the variability of surgical practice due to the relatively large number of surgeons. While indications for surgery and surgical practice were largely consistent, there may have been differences in surgical practice, such as levels of instrumentation and choices on whether to include adjacent affected levels into the construct.

## 5 Conclusion

Since Patchell’s landmark study in 2005, surgical treatment has become the treatment of choice for MESCC and spinal instability. While there has been no change in tumor epidemiology there has been an increase in the surgical treatment of spinal metastases at our center. Minimally invasive techniques and separation surgery for spinal metastases have come to the forefront, with resultant decreases in blood loss and allogenic transfusion and improvement in neurological and ambulatory outcomes. While patient survival has improved at 1 and 3 months, surgery does not improve overall survival. The decision for surgery should be made in a multidisciplinary manner and even patients with poor survival prognosis and poor performance scores should not be as excluded as they may significantly benefit from surgery.

## Data availability statement

The raw data supporting the conclusions of this article will be made available by the authors, without undue reservation.

## Ethics statement

The studies involving humans were approved by NHG Domain Specific Review Board. The studies were conducted in accordance with the local legislation and institutional requirements. Written informed consent for participation was not required from the participants or the participants’ legal guardians/next of kin in accordance with the national legislation and institutional requirements.

## Author contributions

JT: Conceptualization, Data curation, Formal analysis, Funding acquisition, Investigation, Methodology, Project administration, Resources, Software, Supervision, Validation, Visualization, Writing – original draft, Writing – review & editing. JH: Conceptualization, Data curation, Formal analysis, Funding acquisition, Investigation, Methodology, Project administration, Resources, Software, Supervision, Validation, Visualization, Writing – original draft, Writing – review & editing. RL: Data curation, Formal analysis, Investigation, Methodology, Project administration, Resources, Visualization, Writing – original draft. YC: Conceptualization, Formal analysis, Funding acquisition, Methodology, Resources, Software, Visualization, Writing – original draft, Writing – review & editing. TT: Data curation, Formal analysis, Investigation, Methodology, Project administration, Resources, Visualization, Writing – original draft. SA: Data curation, Formal analysis, Investigation, Methodology, Project administration, Resources, Visualization, Writing – original draft. LT: Data curation, Formal analysis, Investigation, Methodology, Project administration, Resources, Visualization, Writing – original draft. JWIT: Data curation, Formal analysis, Investigation, Methodology, Project administration, Resources, Visualization, Writing – original draft. QS: Data curation, Formal analysis, Investigation, Methodology, Project administration, Resources, Visualization, Writing – original draft. DH: Data curation, Formal analysis, Investigation, Methodology, Project administration, Resources, Supervision, Validation, Visualization, Writing – original draft. LL: Data curation, Formal analysis, Investigation, Methodology, Project administration, Resources, Supervision, Validation, Visualization, Writing – original draft. JT: Data curation, Formal analysis, Investigation, Methodology, Project administration, Resources, Supervision, Validation, Visualization, Writing – original draft. HW: Data curation, Formal analysis, Investigation, Methodology, Project administration, Resources, Supervision, Validation, Visualization, Writing – original draft. GL: Data curation, Formal analysis, Investigation, Methodology, Project administration, Resources, Supervision, Validation, Visualization, Writing – original draft. NK: Conceptualization, Data curation, Formal analysis, Funding acquisition, Investigation, Methodology, Project administration, Resources, Software, Supervision, Validation, Visualization, Writing – original draft, Writing – review & editing.
